# Elevated Risk of Psychotropic Drug Prescriptions in Adults With Mastocytosis: A Danish Matched Cohort Study

**DOI:** 10.1002/clt2.70223

**Published:** 2026-11-15

**Authors:** Maren Poulsgaard Jørgensen, Andreas Kiesbye Øvlisen, Jonas Faartoft Jensen, Helene Bjørg Kristensen, Tarec Christoffer El‐Galaly, Hanne Vestergaard, Sigurd Broesby‐Olsen, Marianne Tang Severinsen

**Affiliations:** ^1^ Department of Hematology Clinical Cancer Research Center Aalborg University Hospital Gistrup Denmark; ^2^ Department of Clinical Medicine Aalborg University Gistrup Denmark; ^3^ Department of Hematology Aarhus University Hospital Aarhus Denmark; ^4^ Department of Hematology Odense University Hospital Odense Denmark; ^5^ Mastocytosis Centre Odense University Hospital (MastOUH) Odense Denmark; ^6^ Department of Dermatology and Allergy Centre Odense University Hospital Odense Denmark

**Keywords:** anxiety, depression, mastocytosis, mental health, psychotropic drug

## Abstract

**Background:**

Mastocytosis is a clonal mast cell disorder characterized by chronic mediator‐related symptoms and heterogeneous systemic involvement. Neuropsychiatric symptoms are frequently reported, yet population‐based evidence on incident depression and/or anxiety remains limited.

**Objective:**

To examine the mental health burden among patients with mastocytosis.

**Methods:**

We conducted a population‐based matched cohort study using Danish health registers to evaluate incident, pharmacologically treated depression/anxiety among adult patients diagnosed with mastocytosis between 2005 and 2023. Patients were matched 1:5 with individuals from the general Danish population on age, sex, Charlson Comorbidity Index, and educational level. Individuals with psychotropic drug (PD) prescription within 10 years prior to index were excluded. Cumulative risks were estimated using the Aalen‐Johansen estimator, and hazard ratios (HRs) were calculated using Cox proportional hazard regression.

**Results:**

We included 874 patients with mastocytosis and 4370 comparators. Patients had a higher risk of incident PD prescription in the 10 years following diagnosis (*p* < 0.001). The 5‐year cumulative risk was 13.1% in patients and 8.4% in comparators. The HR for incident PD use was 1.72 (95% confidence interval 1.38–2.15, *p* < 0.001). Results were consistent across sensitivity and landmark analyses. Higher age, comorbidity, and shorter education were associated with increased risk.

**Conclusion:**

Mastocytosis was associated with an increased risk of PD prescriptions, possibly reflecting an increased burden of pharmacologically treated depression and/or anxiety, implying the importance of mental health awareness in clinical management.

## 1 Introduction

Mastocytosis is a group of heterogeneous chronic neoplasms that affect the skin and/or extra‐cutaneous organs [1, 2]. The majority have an indolent course (non‐advanced systemic mastocytosis, non‐AdvSM) with life expectancy similar to the general population. Few patients with systemic mastocytosis (SM) have an aggressive course (advanced SM, AdvSM) with short‐term survival [3, 4]. Mastocytosis is characterized by mutations in the proto‐oncogene c‐*KIT* [5, 6, 7] resulting in clonal proliferation and constitutive activation of mast cells, leading to the release of cytokines and inflammatory mediators [7, 8]. The release of mediators within the central nervous system [9, 10, 11] may promote neuroinflammatory processes [11, 12, 13], and have been implicated in the pathophysiology of depression [14, 15]. Previous studies have reported depression prevalence rates of 50%–60% across different mastocytosis cohorts [16, 17, 18].

Worldwide, depression and anxiety are the most prevalent psychiatric disorders and represent a major and growing public health concern [19]. Beyond their psychological impact, depression and anxiety are associated with impaired socioeconomic functioning, reduced quality of life, and increased morbidity and mortality [20, 21]. Depression and anxiety frequently co‐occur with cancer and chronic diseases, reflecting the substantial psychological and biological burden associated with living with a chronic and/or life‐threatening condition [22, 23, 24, 25, 26, 27]. Increasing evidence suggests that immune dysregulation and systemic inflammation may contribute to the link between chronic disease and psychiatric disorders [28, 29].

The aim of this population‐based study was to examine the mental health burden after diagnosis of mastocytosis among adult Danish patients compared to a matched cohort from the general population. We used redeemed psychotropic drug (PD) prescriptions, contact with psychiatric departments, contact with tax‐funded private psychiatrists/psychologists, and intentional self‐harm or suicide attempts as proxies for depression and anxiety.

## 2 Methods

### 2.1 Setting

This population‐based matched cohort study used Danish health registers to investigate mental health in patients with mastocytosis compared to the general population. All residents are registered in the Danish Civil Registration System, which includes sex, birth date, death date, and a unique personal identification number enabling individual‐level linkage across registers and complete long‐term follow‐up [30, 31, 32, 33]. The tax‐supported health care system in Denmark provides universal access, and healthcare contacts are systematically recorded [34, 35]. However, diagnoses made in general practice are not recorded. As depression and anxiety are primarily managed in this setting [36], redeemed prescriptions for PDs were used as proxies for psychiatric morbidity. These medications are typically prescribed for severe depression or for moderate depression when psychotherapy alone is insufficient [37, 38]. Data on all redeemed prescriptions were obtained from the Danish National Prescription Registry (DNPR), providing comprehensive information on pharmacological treatment.

### 2.2 Study Population

The patient cohort consisted of adult patients (≥ 18‐year‐old) diagnosed with mastocytosis in Denmark between January 1, 2005, and December 31, 2023. Patients were identified by merging information from the Danish National Patient Register, the Danish Pathology Register, and the Danish Cancer Register as previously outlined in detail [39]. A diagnosis of mastocytosis was considered valid by either (1) biopsy‐verification, (2) ≥ two International Classification of Diseases, 10th revision (ICD‐10) codes over time for mastocytosis given by a dermatologist, hematologist, or allergist, or (3) a single ICD‐10 code for mastocytosis given by a dermatologist, hematologist, or allergist combined with a positive KIT mutation and/or high tryptase level ≥ 20 ng/mL. Mastocytosis subtypes were categorized as cutaneous mastocytosis (CM), mastocytosis in the skin (MIS), non‐AdvSM, and AdvSM. A provisional diagnosis of MIS was used when no extra‐cutaneous biopsy confirmed SM and no bone marrow biopsy had been performed; thus, SM could neither be confirmed nor excluded. The index date was the first date of any mastocytosis diagnosis.

Each patient was matched with five individuals from the general Danish population on sex, birth year, Charlson Comorbidity Index (CCI), and educational level, excluding those with prior mastocytosis. Exact matching was used for sex, birth year, and educational level. For CCI, matching was exact when possible; otherwise, patients were matched within predefined intervals. No patients were excluded due to lack of suitable comparators. Five controls were selected to improve statistical power. Previous methodological studies have demonstrated that there is limited additional power gained by increasing the matching ratio beyond 1:4 or 1:5 [40, 41]. The index date for comparators corresponded to the diagnosis date for the matched patient. The CCI was calculated 6 months before the index date using data from the Danish National Patient Register and the DNPR [42, 43]. Highest attained educational level at index was identified from the Danish Education Register using International Standard Classification of Education (ISCED) codes [33, 44] and categorized according to SEPLINE guidelines as short (ISCED 1–2; approximately 9–10 years of education), medium (ISCED 3; approximately 12–13 years) or long (ISCED 5–8; tertiary education exceeding 13 years). ISCED 4 is not used in Denmark [33].

To reduce the likelihood that patients and matched comparators had a prior history of depression or anxiety, individuals with any of the following within 10 years preceding the index date were excluded: (1) PD prescriptions (antidepressants, antipsychotics, or anxiolytics); (2) contact with a psychiatric department for depression or anxiety; or (3) contact with a tax‐funded private psychiatrist/psychologist. The 10‐year look‐back period reflected the maximum available period in the registers.

### 2.3 Outcome Definitions

The primary outcome was first redeemed PD prescription after index. Secondary outcomes included (a) first in‐/outpatient contact for depression or anxiety, (b) first contact with a tax‐funded private psychiatrist/psychologist, and (c) first incident of intentional self‐harm or completed suicide. PD prescriptions were identified from the DNPR, which records all redeemed prescriptions since 1994 using Anatomical Therapeutic Chemical (ATC) codes [45, 46]. Prescription indications have been available since 2004, although with varying completeness [36]. In this study, antidepressants (ATC N06A), antipsychotics (ATC N05A), and anxiolytics (ATC N05B) were used as proxies for depression and anxiety. In sensitivity analyses, outcomes were restricted to antidepressants with a recorded indication of depression or anxiety. Further, bupropion (ATC N06AX12) and hydroxyzine (ATC N05BB01) were excluded due to their use in smoking cessation and allergic skin reactions, respectively. Prescriptions for hypnotics (ATC N05C) were examined as an alternative indicator of mental distress. Finally, a second redeemed PD prescription was assessed to better capture persistent psychiatric morbidity.

Landmark analyses at 1, 2, and 5 years after diagnosis were conducted among patients alive and without prior PD prescriptions, with rematching to five new comparators at each time point. To assess whether PD prescriptions preceded the diagnosis of mastocytosis, we analyzed first PD prescription from 5 years before to 5 years after diagnosis, allowing prior PD prescriptions up to 5 years before diagnosis. The index date was redefined to 5 years before diagnosis and patients were rematched with individuals who survived at least 5 years from the new index date.

Psychiatric hospital contacts for depression or anxiety were identified from the Danish Psychiatric Central Research Register using ICD‐10 codes [47]. Intentional self‐harm and suicides were identified using a modified DK‐algorithm [48, 49] (Supporting Information S1: Table S1). Contacts with tax‐funded private psychiatrist/psychologists were identified from the Danish National Health Insurance Service Register. These practitioners are private but contracted with the public healthcare system, therefore treatment can be fully tax‐funded, fully self‐paid, involve co‐payment, or financed through private insurance. Only publicly reimbursed services are included in the register, but diagnoses are not recorded [50].

### 2.4 Statistical Analyses

Continuous variables are presented as medians with interquartile ranges (IQRs) and categorical variables as frequencies and percentages. The follow‐up period was defined as the time from the index date until the occurrence of any of the outcome variables, death from any cause, later mastocytosis diagnosis among comparators, or censoring (emigration or end of follow‐up on December 31, 2024), whichever came first.

Cumulative incidence of the mental health outcome variables was estimated using the Aalen‐Johansen estimator, with death and a later mastocytosis diagnosis (among comparators) treated as competing events and with pointwise 95% confidence intervals (CI) included. In the primary analysis, cumulative incidence of any PD (including stratification by type of PD) during the 10 years following index was estimated and compared between patients and comparators using Gray's test [51]. Similar comparisons were conducted in both secondary, landmark, and sensitivity analyses described previously.

Cox proportional hazards models were used to estimate 5‐year cause‐specific hazard ratios (HRs) for any PD prescription, with follow‐up censored at 5 years after index in case of no event. Two analytical setups were applied. First, comparisons between patients and comparators were conducted overall and stratified by age, sex, educational level, and CCI, with mastocytosis as the exposure. To account for potential homogeneity of outcomes between mastocytosis patients and comparators within the same matched cluster, a cluster‐robust sandwich estimate of the standard error of each HR was applied [52]. Second, within the mastocytosis cohort, risk factors for PD prescriptions were assessed, including the same covariates with the addition of mastocytosis subtype. In this analysis, associations were estimated using inverse probability weighting to adjust for age and sex [53]. The proportional hazards assumption was evaluated using visual inspection of Schoenfeld residuals. Hazard ratios were reported with 95% CIs and Wald test *p*‐values.

*p*‐values < 0.05 were considered significant. Median follow‐up was estimated using the reverse Kaplan‐Meier estimator. The statistical analyses were performed using SAS version 9.4 (SAS Institute Inc., Gary, North Carolina, USA) and R statistical software (version 4.4.0, R Foundation for Statistical Computing, Vienna, Austria). The project was approved by the Region North, Denmark (F2022‐009).

## 3 Results

### 3.1 Baseline Characteristics

A total of 874 (402 non‐AdvSM [46.0%], 271 MIS [31.0%], 112 AdvSM [12.8%], and 89 CM [10.2%]) patients and 4370 comparators were identified (Supporting Information S1: Figure S1). Median age of the patients was 52 years (IQR 40–67 years) and 51.4% were males (Table 1). Median follow‐up was 7.5 years (7.2–7.8 years). The excluded patients (*n* = 449, 34%) were slightly older (56 years, [IQR 44–68]), predominantly female (64.1%) with a higher prevalence of comorbidity (41.9%) and lower educational level (short level, 27.5%) compared to the included patients (Supporting Information S1: Table S2).

**TABLE 1:** Baseline characteristics of included patients with mastocytosis and matched comparators.

	Mastocytosis patients *N* = 874	Matched comparators *N* = 4370
Age at index date, median (IQR)	52 (40–67)	52 (39–67)
Sex, *n* (%)		
Female	425 (48.6%)	2125 (48.6%)
Male	449 (51.4%)	2245 (51.4%)
Charlson comorbidity index, *n* (%)		
0	662 (75.7%)	3310 (75.7%)
≥ 1	212 (24.3%)	1060 (24.3%)
Educational level, *n* (%)		
Short education	163 (19.1%)	815 (19.1%)
Medium education	369 (43.2%)	1845 (43.2%)
Long education	322 (37.7%)	1610 (37.7%)
Missing information	20	100
Marital status		
Unmarried	227 (26.0%)	1201 (27.5%)
Widowed	48 (5.5%)	213 (4.9%)
Divorced	80 (9.2%)	461 (10.5%)
Married	519 (59.4%)	2495 (57.1%)
Mastocytosis subgroup		
CM	89 (10.2%)	—
MIS	271 (31.0%)	—
Non‐AdvSM	402 (46.0%)	—
AdvSM	112 (12.8%)	—
Tryptase (ng/mL), median (IQR)	19 (9–43)	—
Missing information	263	
Tryptase ≥ 20 ng/mL, *n*(%)		
Yes	349 (57.1%)	—
No	262 (42.9%)	—
Missing information	263	—

Abbreviations: AdvSM: advanced systemic mastocytosis (i.e., aggressive systemic mastocytosis, systemic mastocytosis with an associated hematologic neoplasm, and mast cell leukemia), CM: cutaneous mastocytosis, IQR: inter quartile range, MIS: mastocytosis in the skin, non‐AdvSM: non‐advanced systemic mastocytosis (i.e., indolent systemic mastocytosis, bone marrow mastocytosis, and smoldering systemic mastocytosis).

### 3.2 Incident Psychotropic Drug Prescriptions

During the 10 years following index, 141 patients and 476 comparators redeemed a PD prescription. Of these, 95 and 347 received an antidepressant prescription, 42 and 144 received an anxiolytic prescription, and 25 and 115 received an antipsychotic prescription, respectively (Supporting Information S1: Table S3). The risk of any PD prescription was significantly higher among patients with mastocytosis than comparators during the 10 years after diagnosis (*p* < 0.001) (Figure 1). The overall 5‐year cumulative risk of incident PD prescriptions was 13.1% (95% CI, 10.7%–15.5%) among patients and 8.4% (95% CI, 7.5%–9.3%) among comparators. This corresponded to an overall 5‐year HR of 1.72 (95% CI, 1.38–2.15, *p* < 0.001). Stratified analyses of baseline characteristics were generally consistent with this finding (Table 2). Antidepressants were most frequently prescribed with a 5‐year cumulative risk of 8.5% (95% CI, 6.5%–10.4%) and 6.2% (95% CI, 5.4%–7.0%) in patients and comparators, respectively. Both antidepressant and anxiolytic prescriptions were significantly more common among patients than comparators (*p* = 0.0045 and *p* = 0.028, respectively), whereas no difference was found in antipsychotic prescriptions (*p* = 0.72) (Figure 1).

**FIGURE 1:**
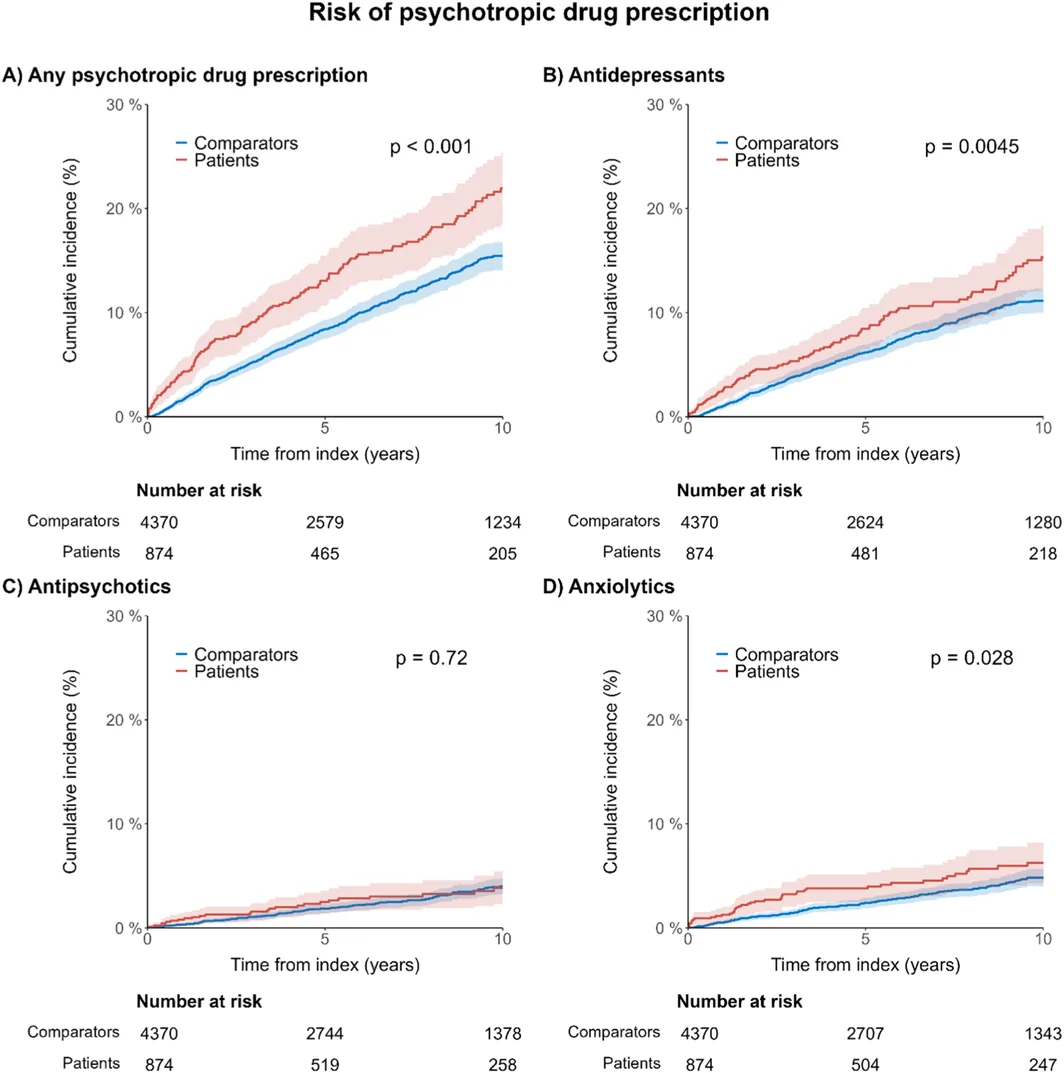
Risk of redeemed psychotropic drug prescriptions among patients with mastocytosis and matched comparators from date of diagnosis of the index patient.

**TABLE 2:** Estimated 5‐year hazard ratios of the association between psychotropic drug prescriptions in patients with mastocytosis and demographic variables relative to the matched comparators (reference), using Cox proportional hazard models.

	HR (95% CI)	*p*‐value
Overall	1.72 (1.38–2.15)	< 0.001
Age		
18–44	1.74 (1.15–2.65)	0.009
45–60	1.30 (0.80–2.12)	0.283
≥ 61	2.04 (1.49–2.80)	< 0.001
Sex, *n* (%)		
Female	1.70 (1.24–2.33)	0.001
Male	1.74 (1.28–2.38)	< 0.001
Educational level (ISCED), *n* (%)		
Short	1.89 (1.26–2.83)	0.002
Medium	1.73 (1.21–2.46)	0.003
Long	1.45 (0.94–2.24)	0.097
CCI, *n* (%)		
0	1.79 (1.35–2.36)	< 0.001
≥ 1	1.67 (1.15–2.41)	0.007

Abbreviations: CCI: Charlson Comorbidity Index, CI: confidence interval, HR: hazard ratio.

The analysis restricted to antidepressant prescriptions with an indication of depression or anxiety did not demonstrate a statistically significant difference between the groups (*p* = 0.094). However, when unspecified neurotic disorder was included as an indication, the difference reached statistical significance (*p* = 0.025) (Supporting Information S1: Figure S2). In all, 52.6% (*n* = 50) and 58.5% (*n* = 203) of antidepressant prescriptions among patients and comparators, respectively, were recorded with an indication of depression or anxiety. Neuropathic pain was the indication in 4.2% and 7.2% of antidepressant prescriptions, respectively. The amount of antidepressant prescriptions with missing/unspecified indications were 29.5% and 20.2%, respectively (Supporting Information S1: Table S4).

Sertraline was the most prescribed antidepressant both among patients (27.4%) and comparators (28.0%) followed by mirtazapine (23.2% and 21.0%, respectively) and citalopram (14.7% and 17.29, respectively) (Supporting Information S1: Table S5). Among anxiolytics, oxazepam was most prescribed (31.0% of patients and 51.4% of comparators) followed by diazepam (26.2% and 22.2%, respectively) and hydroxyzine (23.8% and 6.3%, respectively) (Supporting Information S1: Table S6). After exclusion of hydroxyzine no difference in anxiolytic prescriptions was found between patients and comparators (*p* = 0.42); whereas the exclusion of bupropion did not change the results of antidepressants (*p* = 0.0035) (Supporting Information S1: Figure S3).

In the analysis of redeemed hypnotic prescriptions, a significantly higher cumulative incidence was found among patients with mastocytosis than comparators (*p* = 0.041, 5‐year cumulative risk 8.9% [95% CI, 6.8%–11.0%] and 5.6% [95% CI, 4.8%–6.3%], respectively) (Supporting Information S1: Figure S4).

The analysis of risk of a second PD prescription demonstrated significantly higher risk of any PD prescription (*p* = 0.0037) and antidepressant prescriptions (*p* = 0.0026) among patients, whereas the risk of a second anxiolytic or antipsychotic prescription did not differ between the groups (*p* = 0.37 and *p* = 0.97 respectively) (Supporting Information S1: Figure S5).

### 3.3 Landmark Analysis of Psychotropic Drug Prescriptions

The cumulative risk of incident PD prescriptions remained significantly higher among patients with mastocytosis compared to comparators over time (*p*‐values from < 0.001 to 0.0061) (Figure 2). From 1 year after diagnosis, the 5‐year cumulative risk was 12.3% (95% CI, 9.7%–14.9%) and 7.6% (95% CI, 6.7%–8.5%) among patients with mastocytosis and comparators, respectively. From 2 years after diagnosis, the 5‐year cumulative risk was 10.3% (95% CI, 7.7%–12.9%) and 7.6% (95% CI, 6.6%–8.6%), respectively. Lastly, from 5 years after diagnosis, the 5‐year cumulative risk was 10.8% (95% CI, 7.4%–14.2%) and 5.4% (95% CI, 4.3%–6.5%), respectively.

**FIGURE 2:**
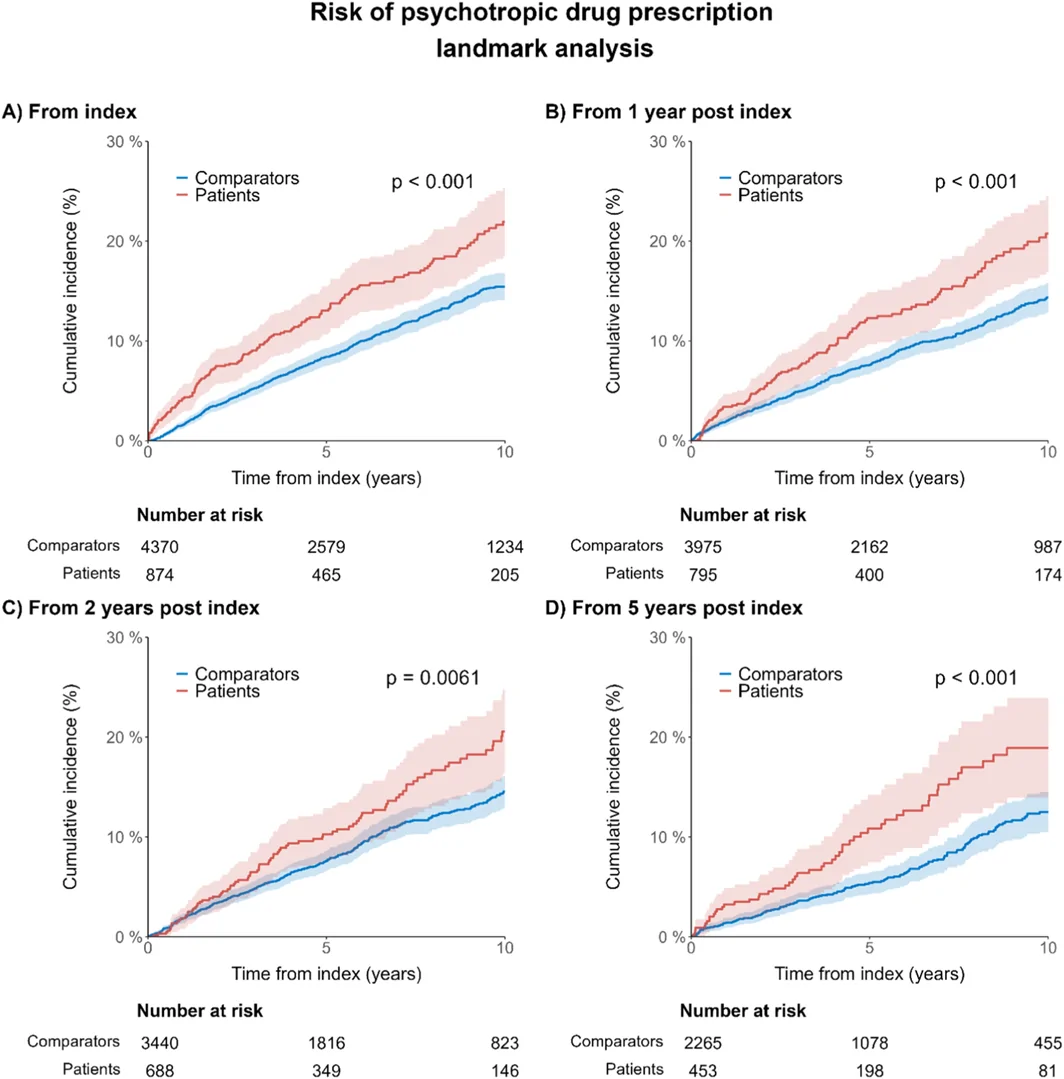
Landmark analysis for the risk of psychotropic drug prescription among patients with mastocytosis and matched comparators at 1, 2, and 5 years after index.

### 3.4 Pre‐Diagnostic Risk of Psychotropic Drug Prescription

An additional analysis examining the timing of incident PD prescription from 5 years before to 5 years after diagnosis demonstrated that the risk of anxiolytic prescription diverged slightly from the comparators in the year before diagnosis, whereas antidepressant‐ and antipsychotic prescriptions did not differ prior to diagnosis. In the months and years following diagnosis, patients deviated further from the matched comparators in the risk of any PD‐, antidepressant‐, and anxiolytic prescriptions (Figure 3). The exclusion of hydroxyzine from this analysis did not alter the results substantially (Supporting Information S1: Figure S6).

**FIGURE 3:**
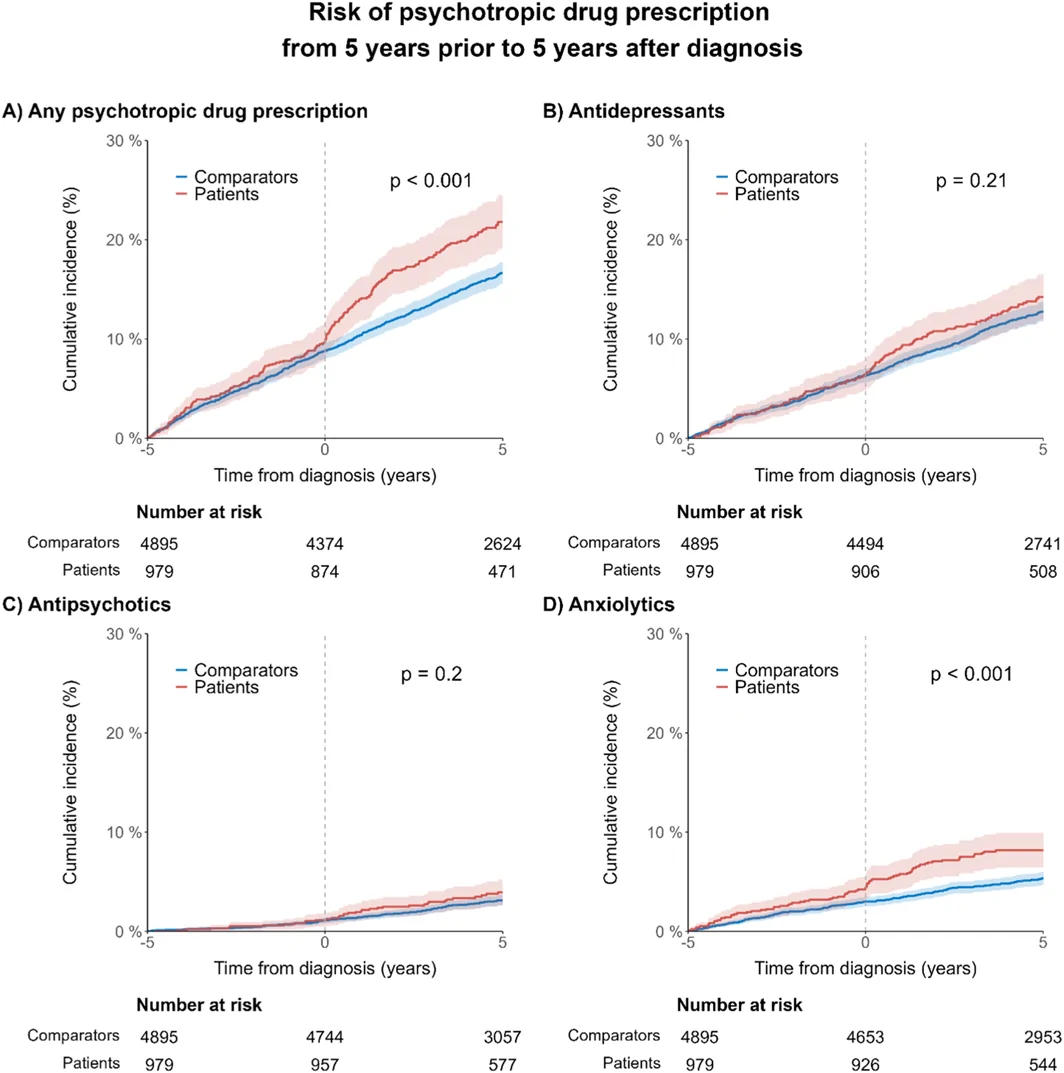
Risk of redeemed psychotropic drug prescriptions among patients with mastocytosis and matched comparators from 5 years before to 5 years after diagnosis.

### 3.5 Risk Factors for Psychotropic Drug Prescriptions

The risk of any PD prescription was higher in patients with higher age, CCI ≥ 1, and short‐length education (Table 3). Subtype of mastocytosis was not associated with PD prescriptions in general nor with antidepressant prescriptions specifically (Table 3 and Supporting Information S1: Table S7). However, the risk of anxiolytic prescriptions was significantly higher in patients with AdvSM compared to patients with CM (HR 13.8 [95% CI, 1.5–125.7], *p* = 0.02) (Supporting Information S1: Table S8).

**TABLE 3:** Risk factor analysis for the association between demographical and clinical baseline characteristics and any psychotropic drug prescriptions among the patients with mastocytosis; based on estimated 5‐year hazard ratios using adjusted Cox proportional hazards models with inverse probability weighting.

	HR (95% CI)	*p*‐value
Age^a^	1.02 (1.01–1.04)	< 0.001
Sex, *n* (%)^a^		
Female	Ref	
Male	0.96 (0.65–1.42)	0.848
Mastocytosis subgroup, *n* (%)^a^		
CM	Ref	
MIS	0.69 (0.32–1.49)	0.346
Non‐AdvSM	0.89 (0.44–1.81)	0.752
AdvSM	2.07 (0.75–5.68)	0.158
Educational level (ISCED), *n* (%)^a^		
Short	Ref	
Medium	0.51 (0.32–0.82)	0.006
Long	0.41 (0.24–0.68)	< 0.001
CCI at diagnosis, *n* (%)^a^		
0	Ref	
≥ 1	1.89 (1.14–3.12)	0.013
Tryptase ≥ 20 ng/mL, *n* (%)^b^		
No	Ref	
Yes	1.33 (0.80–2.21)	0.266

Abbreviations: AdvSM: advanced systemic mastocytosis (i.e., aggressive systemic mastocytosis, systemic mastocytosis with an associated hematologic neoplasm, and mast cell leukemia), CCI: Charlson Comorbidity Index, CI: confidence interval, CM: cutaneous mastocytosis, HR: hazard ratio, MIS: mastocytosis in the skin, non‐AdvSM: non‐advanced systemic mastocytosis (i.e., indolent systemic mastocytosis, bone marrow mastocytosis, and smoldering systemic mastocytosis).

^a^ Adjusted for sex and age.

^b^ Adjusted for sex, age and mastocytosis subtype.

We did not observe substantial differences in PD use according to anti‐mediator treatment during follow‐up. PD use was more frequent among patients treated with leukotriene receptor antagonists or H2‐receptor antagonists than among non‐users, whereas no notable differences were observed for the other anti‐mediator treatments (Supporting Information S1: Table S9).

### 3.6 Incidence of Other Mental Health Complications

In the analysis investigating in‐/outpatient contacts with psychiatric hospital departments regarding depression or anxiety no difference was found between patients with mastocytosis (5‐year cumulative risk 0.5% [95% CI, 0.0%–1.0%]) and comparators (5‐year cumulative risk 0.6% [95% CI, 0.3%–0.8%], *p* = 0.28) (Supporting Information S1: Figure S7A). Similar results were found when contacts were not restricted to depression or anxiety (*p* = 0.079) (Supporting Information S1: Figure S8).

Furthermore, no difference was observed between patients and comparators regarding contacts with private tax‐funded psychiatrists/psychologists (*p* = 0.73, 5‐year cumulative risk 3.9% [95% CI, 3.2%–4.5%] and 3.5% [95% CI, 2.2%–4.8%], respectively) (Supporting Information S1: Figure S7B).

The analysis of intentional self‐harm and completed suicides yielded small numbers of events. No statistically significant difference was found between patients with mastocytosis and comparators (*p* = 0.318) (Supporting Information S1: Figure S7C).

## 4 Discussion

This population‐based matched cohort study includes 874 patients with mastocytosis diagnosed between 2005 and 2023, and 4370 comparators. Our findings demonstrate that the incidence of a PD prescription over a 10‐year period following the index date was significantly higher among patients than comparators. The 5‐year cumulative incidence was 13.1% among patients and 8.4% among comparators, corresponding to a HR of 1.72. The 5‐year risk difference was 4.7% and remained consistent in analyses of second PD prescriptions and among patients who were PD‐naïve up to 5 years after diagnosis. Collectively, these findings may indicate that pharmacologically treated depression and/or anxiety are more common among patients with mastocytosis than in the general population, consistent with previous studies [54, 55]. However, we did not observe an increased risk of psychiatric hospital contacts or self‐harm or suicide, suggesting that the increased use of psychotropic medication does not appear to be accompanied by an increased risk of severe psychiatric morbidity or suicidal behavior. Risk factors for PD prescriptions were older age, comorbidity, and short educational level. We did not observe any difference in antidepressant use across mastocytosis subtypes, whereas AdvSM was associated with an increased rate of anxiolytic prescriptions. However, this finding should be considered exploratory and interpreted with caution given the wide CI, reflecting substantial uncertainty in the estimated association.

Although no prior studies have investigated PD use in patients with mastocytosis, questionnaire‐based studies report a prevalence of depressive symptoms in 50%–75% of patients [17, 18, 54, 56, 57], primarily conducted using the Hamilton Depression Rating Scale (HAM‐D17) or the Beck Depression Inventory. The use of questionnaires in this setting has limitations, as somatic symptoms of mast cell activation may be misinterpreted as depressive symptoms, potentially overestimating the burden of depression [58]. Furthermore, diagnostic thresholds used to define depression vary across studies. Hermine et al. defined depression as a HAM‐D17 score ≥ 10 [54], while Moura et al., defined mild‐to‐moderate depression as 8–22 and severe depression as a ≥ 23 [56], and Jendoubi et al. defined mild, moderate, and severe depression as 8–16, 17–23, and ≥ 24, respectively [17]. By contrast, Danish guidelines classify 13–17 as mild depression, 18–24 as moderate, and ≥ 25 as severe [38]. The lower cut‐offs used in these studies likely resulted in a greater proportion of patients classified with mild depression compared with clinical practice. We observed a 5‐year cumulative incidence of PD prescriptions of 13.1% among patients, possibly reflecting patients with moderate‐to‐severe depression, as mild depression typically does not require pharmacological treatment. This aligns with the findings by Jendoubi et al., where 13% of patients had moderate‐to‐severe depression [17]. In contrast, Georgin‐Lavialle et al. reported a higher prevalence of moderate‐to‐severe depression (29.6%) using the Beck Depression Inventory (score ≥ 19) [18]. The difference may partly reflect differences in sex distribution, as female sex is generally associated with a higher incidence of depression [59, 60]. The proportion of women was higher in the study by Georgin‐Lavialle et al. (66.7%) than in the present study (48.6%) and that by Jendoubi et al. (56.1%). Another explanation for the differences is that the present study investigated incident use rather than prevalent cases, as is typical in questionnaire‐based studies. This approach was chosen to evaluate whether a diagnosis of mastocytosis is associated with an increased risk of subsequent PD prescriptions, whereas studies of prevalent cases reflect overall burden but limit inference on temporality and causality between mastocytosis diagnosis and initiation of PD treatment. Accordingly, patients with PD use within 10 years before diagnosis were excluded, corresponding to the maximum look‐back period available in the DNPR. This resulted in 34% of the patients being excluded. These excluded patients were slightly older (4 years older), predominantly female (64.1%), and more likely to have a short educational level (27.5%), which are all well‐known risk factors for depression and anxiety [61, 62]. While this exclusion increases the likelihood that observed PD use is attributable to mastocytosis rather than pre‐existing psychiatric morbidity, it also introduces a degree of selection into the study population. Individuals with previous psychiatric morbidity, who may be at higher risk of subsequent depression or anxiety, are not represented. Consequently, the absolute 5‐year risks reported in this study may underestimate the risk that would be observed in an unselected population. However, because the same exclusion criteria were applied to the matched comparators, the relative comparison between patients and comparators is less likely to be affected by this selection.

Mastocytosis is often associated with diagnostic delay. Previous studies have reported a median of 2–10 years from symptom onset to diagnosis [4, 63, 64, 65, 66], during which unpredictable symptoms and diagnostic uncertainty may adversely affect mental health [54, 55, 67]. Thus, psychological distress related to mastocytosis may [9] precede the formal diagnosis, and some patients may initiate PD treatment before diagnosis. However, analyses of PD use during the 5 years preceding diagnosis showed that most treatment initiation occurred after the diagnosis, providing some reassurance that this did not substantially influence the results. The increase in PD use after diagnosis may have several explanations. Receiving a diagnosis of a chronic disease, including awareness of the potential severity and risk of anaphylaxis, may itself contribute to psychological distress. Alternatively, the diagnostic process may increase contact with healthcare professionals familiar with mastocytosis, facilitating recognition and treatment of previously unrecognized mental health symptoms. However, the observational design of this study does not allow us to determine the underlying cause of the increased PD use.

Analyses restricted to prescriptions with a recorded indication of depression or anxiety showed no difference between groups. However, 20%–30% of prescriptions had a missing/unspecified indication, which may have reduced statistical power and complicated interpretation. The observed association when prescriptions with an unspecified neurotic disorder indication were included supports this interpretation. Consequently, the primary outcome may partly reflect broader psychological distress rather than depression and/or anxiety specifically, introducing uncertainty in its interpretation. Nevertheless, a true lack of association cannot be excluded. Antidepressants such as selective serotonin reuptake inhibitors and tricyclic antidepressants may also be prescribed for mastocytosis‐related symptoms such as pruritus, neuropathic pain, or sleep disturbances, potentially resulting in differential misclassification. However, individual drug classes did not show increased prescriptions of medications commonly used for these alternative indications (i.e., amitriptyline and fluoxetine) [68, 69, 70], suggesting that such misclassification is unlikely to fully explain the observed findings. In contrast, the increased risk of anxiolytic prescriptions should be interpreted with caution, as the association disappeared after exclusion of hydroxyzine (*p* = 0.42). More patients than comparators had received hydroxyzine for pruritus, consistent with its use for mastocytosis‐related symptoms (data not shown due to data protection regulation), In addition, the increased risk was not sustained when examining the risk of a second anxiolytic prescription, further suggesting that the finding may reflect short‐term or symptoms‐related treatment. Somatic manifestations of mastocytosis, such as tachycardia and facial flushing, may occasionally have been misinterpreted as anxiety, resulting in short‐term anxiolytic treatment. In contrast, the increased risk of antidepressant prescriptions persisted for a second prescription, suggesting a more sustained treatment pattern.

Depression is associated with an increased risk of self‐harm and suicide [60]. Although patients with mastocytosis had a higher rate of PD prescriptions, we did not observe differences in intentional self‐harm, suicide, psychiatric contacts, or contacts with private psychiatrists/psychologists. These findings should be interpreted with caution given the small number of self‐harm and suicide events and the incomplete capture of privately funded psychiatric/psychological care. Nevertheless, our findings from the secondary analyses do not support an association between mastocytosis and severe psychiatric morbidity requiring specialized care, suggesting that any increased psychological burden is more likely to be moderate in severity.

This study has several strengths. To our knowledge, this is the largest study investigating depression and anxiety among patients with mastocytosis. It is based on validated national registers and includes a systematic validation procedure for identifying patients with mastocytosis. The use of the registers minimizes loss to follow‐up and eliminates recall bias. The use of PD prescriptions may better capture psychiatric morbidity than hospital records alone, as most patients are managed in general practice, which is not included in the hospital registers [71, 72]. However, as specific indications were not available for all redeemed PDs, we cannot confirm that all individuals with a PD prescription had depression or anxiety, which may have influenced the results, given that missing/unspecified indications were more frequent among patients. Furthermore, mild cases are generally not treated pharmacologically; therefore, the study reflects an estimate of at least moderate disease severity rather than the full burden. Moreover, the risk of depression and anxiety may also be underestimated, as some individuals do not seek medical attention. The regular healthcare contact among patients with mastocytosis may increase symptom detection and treatment, thereby introducing potential surveillance bias that could overestimate the incidence of pharmacologically treated depression and/or anxiety compared with comparators. Finally, as clinical information on mastocytosis‐related symptoms is not captured in the registers, we were unable to assess whether symptom frequency or severity was associated with the onset of PD use. Further studies incorporating clinical information are warranted to investigate this potential association.

Overall, these findings indicate that patients with mastocytosis are at increased risk of initiating PD treatment, possibly due to an increased risk of depression and anxiety. In a selected cohort of individuals without prior psychiatric morbidity, mastocytosis was associated with a 5‐year cumulative risk of PD prescription of 13.1%, compared with 8.4% in the general population (HR 1.72), with the highest risk observed among older patients, those with greater comorbidity burden, and those with a short educational level. These findings suggest that attention to mental health is warranted in patients with mastocytosis and contribute to the understanding of mental health outcomes in this population.

## Author Contributions

**Maren Poulsgaard Jørgensen:** conceptualization, methodology, formal analysis, writing – review and editing, writing – original draft, funding acquisition. **Andreas Kiesbye Øvlisen:** methodology, writing – review and editing. **Jonas Faartoft Jensen:** methodology, formal analysis, software, writing – review and editing, visualization. **Helene Bjørg Kristensen:** writing – review and editing. **Tarec Christoffer El‐Galaly:** writing – review and editing, methodology. **Hanne Vestergaard:** writing – review and editing. **Sigurd Broesby‐Olsen:** writing – review and editing. **Marianne Tang Severinsen:** writing – review and editing, conceptualization, methodology, project administration, supervision.

## Funding

Aase and Ejnar Danielsen's foundation.

## Ethics Statement

This project was carried out as register‐based cohort study without direct contact with patients; hence, no approval from the Danish ethical committee is needed. The project is approved by the Region North, Denmark (F2022‐009).

## Conflicts of Interest

The authors declare no conflicts of interest.

## Supporting information


Supporting Information S1

## Data Availability

The project was carried out on a secured server at Statistics Denmark, for which only a limited number of scientists are allowed access to ensure the safety of the data. Research data are therefore not shared, as export of data from Statistics Denmark is not allowed.
