# Supplementary material for: Elevated Risk of Psychotropic Drug Prescriptions in Adults With Mastocytosis: A Danish Matched Cohort Study
Source: Clin Transl Allergy. 2026 Nov 15;16(11):e70223. doi: 10.1002/clt2.70223 (PMC13770542; doi:10.1002/clt2.70223)
Supplement: Supplementary file 1 [file CLT2-16-e70223-s001.docx]

## **Supplementary**

**Table S1: Adjusted DK-algorithm for intentional self-harm and suicidal events**

| **List of suicide, suicidal attempts, and self-harm with corresponding relevant codes** | |
| --- | --- |
| **Causes of Death register** | |
| **Variable (death cause)** | **Death type** |
| Suicide | 2 |
| **National Patient Register** | |
| **Variable** | **ICD-10 code** |
| Lesion on elbow or forearm | S517-S519, S59 |
| Lesion of blood vessels in forearm / elbow region | S55 |
| Lesions on wrist | S617-S619, S65, S69 |
| Poisoning/intoxications | T36-T50, T52-T60, T65 |
| Intentional self-harm | DX60-84 |
| **Variable – reason for contact** | **Code** |
| Suicide/suicidal attempt | 4 |
| Suicide | ALCC04 |
| Intentional self-harm | ALCC05 |

To estimate the amount of suicide, suicidal attempts, and intentional self-harm among patients with mastocytosis and matched comparators the DK-algorithm supplemented with information from the Danish Register of Cases of Death on completed suicides was used.

T39, T40, T42, T43, DX60-84 and contact codes 4, ALCC04, and ALCC05 are independently classified as intentional self-harm, whereas the remaining S and T codes in Table S1 must be concomitant with an either preexisting diagnosis of any mental disorder (any F codes) or an F code given within 1 years of the S or T code.

**Table S2:** Characteristics of patients excluded due to psychotropic drug prescription, contact with psychiatric hospital departments, or contact with private tax-funded psychiatrist/psychologist within 10 years prior to diagnosis.

|  | **Mastocytosis patients**  **N = 449** |
| --- | --- |
| **Age at index date, median (IQR)** | 56 (44-68) |
| **Sex, n (%)** |  |
| Female | 288 (64.1%) |
| Male | 161 (35.9%) |
| **Charlson Comorbidity Index, n (%)** |  |
| 0 | 261 (58.1%) |
| ≥1 | 188 (41.9%) |
| **Educational level, n (%)** |  |
| Short education | 120 (27.5%) |
| Medium education | 179 (41.1%) |
| Long education | 137 (31.4%) |
| Missing information | 13 |
| **Civil status** |  |
| Unmarried | 92 (20.5%) |
| Widowed | 34 (7.6%) |
| Divorced | 76 (16.9%) |
| Married | 247 (55.0%) |
| **Mastocytosis subgroup** |  |
| CM | 49 (10.9%) |
| MIS | 165 (36.7%) |
| Non-AdvSM | 179 (39.9%) |
| AdvSM | 56 (12.5%) |
| **Tryptase (ng/ml), median (IQR)** | 20 (8-40) |
| Missing information | 174 |
| **Tryptase ≥ 20 ng/ml, n(%)** |  |
| Yes | 157 (57.1%) |
| No | 118 (42.9%) |
| Missing | 174 |
| Abbreviations: CM: cutaneous mastocytosis, MIS: mastocytosis in the skin, non-AdvSM: non-advanced systemic mastocytosis, AdvSM: advanced systemic mastocytosis | |

**Table S3: Number of events during the 10-year follow-up**

|  | **Patients** | **Comparators** |
| --- | --- | --- |
| All | 141 | 476 |
| Antidepressants | 95 | 347 |
| Antipsychotics | 25 | 115 |
| Anxiolytics | 42 | 144 |

**Table S4: Indications recorded with first redeemed antidepressant prescription**

| **Indications** | **Patients**  **N = 95** | **Comparators**  **N = 347** |
| --- | --- | --- |
| Depression, n (%) | 42 (44.2%) | 168 (48.4%) |
| Anxiety, n (%) | 8 (8.4%) | 35 (10.1%) |
| Unspecified neurotic disorder, n (%) | 8 (8.4%) | 26 (7.5%) |
| Neuropathic pain, n (%) | 4 (4.2%) | 25 (7.2%) |
| Other, n (%) | 5 (5.3%) | 23 (6.6%) |
| Missing/unspecified, n (%) | 28 (29.5%) | 70 (20.2%) |

**Table S5: Types of antidepressants prescribed**

| **Drug** | **Type** | **Patients (n=95)** | **Comparators (n=347)** |
| --- | --- | --- | --- |
| Sertraline | SSRI | 26 (27.37%) | 97 (27.95%) |
| Mirtazapine | NaSSA | 22 (23.16%) | 73 (21.04%) |
| Citalopram | SSRI | 14 (14.74%) | 60 (17.29%) |
| Amitriptyline | TCA | 11 (11.58%) | 52 (14.99%) |
| Duloxetine | SNRI | 5 (5.26%) | 15 (4.32%) |
| Escitalopram | SSRI | 5 (5.26%) | 9 (2.59%) |
| Nortriptyline | TCA | 4 (4.21%) | 6 (1.73%) |
| Other* |  | 8 (8.42%) | 35 (10.09%) |
| Abbreviations: NaSSA: noradrenergic and specific serotonergic antidepressants, SNRI: serotonin-norepinephrine reuptake inhibitor, SSRI: selective serotonin reuptake inhibitor,  TCA: tricyclic antidepressants  *Due to data protection, groups of less than 3 patients are grouped together as “other” | | | |

**Table S6: Types of anxiolytics prescribed**

| **Drug** | **Type** | **Patients (n=42)** | **Comparators (n=144)** |
| --- | --- | --- | --- |
| Oxazepam | Benzodiazepine | 13 (30.95%) | 74 (51.39%) |
| Diazepam | Benzodiazepine | 11 (26.19%) | 32 (22.22%) |
| Hydroxyzine | Diphenylmethane derivate | 10 (23.81%) | 9 (6.25%) |
| Lorazepam | Benzodiazepine | 5 (11.90% | 6 (4.17%) |
| Alprazolam | Benzodiazepine | 3 (7.14%) | 16 (11.11%) |
| Other* |  | 0 (0%) | 7 (4.86%) |
| *Due to data protection, groups of less than 3 patients are grouped together as “other” | | | |

**Table S7: Five-year hazard ratio of antidepressant prescription between mastocytosis subgroups**

|  | | Adjusted | |
| --- | --- | --- | --- |
|  | | HR (95% CI) | p-value |
| Mastocytosis subgroup | |  |  |
|  | CM | ref |  |
|  | MIS | 0.50 (0.20-1.24) | 0.136 |
|  | Non-AdvSM | 0.74 (0.33-1.67) | 0.472 |
|  | AdvSM | 1.05 (0.35-3.16) | 0.927 |
| *Abbreviations: CM: cutaneous mastocytosis, MIS: mastocytosis in the skin, non-AdvSM: non-advanced systemic mastocytosis, AdvSM: advanced systemic mastocytosis* | | | |

**Table S8: Five-year hazard ratio of anxiolytics prescription between mastocytosis subgroups**

|  | | Adjusted | |
| --- | --- | --- | --- |
|  | | HR (95% CI) | p-value |
| Mastocytosis subgroup | |  |  |
|  | CM | ref |  |
|  | MIS | 2.33 (0.29-18.73) | 0.426 |
|  | Non-AdvSM | 2.64 (0.34-20.13) | 0.350 |
|  | AdvSM | 13.81 (1.52-125.67) | 0.020 |
| *Abbreviations: CM: cutaneous mastocytosis, MIS: mastocytosis in the skin, non-AdvSM: non-advanced systemic mastocytosis, AdvSM: advanced systemic mastocytosis* | | | |

**Table S9 Anti-mediator treatment and psychotropic drug use among patients with mastocytosis**

| **Type of anti-mediator treatment** | **ATC code** | **N with medication within 10-year follow-up/events/%** | **N without medication within 10-year follow-up/events/%** |
| --- | --- | --- | --- |
| Any anti-mediator |  | 663/111/16.7 | 211/30/14.2 |
| Systemic antihistamine | R06A | 441/72/16.3 | 433/69/15.9 |
| H2-receptor antagonist | A02BA | 49/13/26.5 | 825/128/15.5 |
| Proton pump inhibitor | A02BC | 303/54/17.8 | 571/87/15.2 |
| Cromolyn | A07EB01 | .../.../18.2* | .../.../16.1* |
| Leukotriene receptor antagonist | R03DC | 21/9/42.9 | 853/132/15.5 |
| NSAID | M01A | 353/59/16.7 | 521/82/15.7 |
| **Due to data protection regulations, data for groups with fewer than three individuals, or groups from which such groups could be identified, are not reported in accordance with Statistics Denmark’s requirements.  Abbreviations: ATC: Anatomical therapeutic chemical, n: number, NSAID: nonsteroidal anti-inflammatory drug | | | |

**Figure S1 Flow chart of included patients with mastocytosis**

Figure S1 Abbreviations: DCR: Danish Cancer Register, DPR: Danish Pathology Register, NPR: Danish National Patient Register

*A diagnosis of mastocytosis was validated by either 1) positive biopsy 2) two or more International Classification of Diseases, 10th revision (ICD-10) codes over time for mastocytosis given by a dermatologist, hematologist, or an allergist, or 3) a single ICD-10 code for mastocytosis given by a dermatologist, hematologist, or an allergist combined with a positive KIT mutation and/or high tryptase level ≥20 ng/ml.

**Figure S2**

Figure S2 Risk of first redeemed antidepressant prescription with an indication of A) depression or anxiety and B) depression, anxiety, or unspecified neurotic disorder among patients with mastocytosis and matched comparators. Greys test was used to investigate differences in cumulative incidences between patients and comparators. The cumulative incidence of prescriptions with an indication of depression or anxiety did not reach statistical significance. The difference was significant with the inclusion of unspecific neurotic disorder as indication.

**Figure S3**

Figure S3: Risk of first redeemed psychotropic drug prescriptions (PD) after exclusion of hydroxyzine and bupropion from the PD definition. Greys test was used to investigate differences in cumulative incidences between patients and comparators. The cumulative incidences of any PD and antidepressant prescription were significantly higher among patients than matched comparators.

**Figure S4**

Figure S4 Risk of first redeemed A) psychotropic drug (PD) prescription (i.e., antidepressant, antipsychotic, anxiolytic and hypnotic) and B) hypnotic prescription among patients with mastocytosis and matched comparators. Greys test was used to investigate differences in cumulative incidences between patients and comparators. The cumulative incidences of any PD- and anxiolytic prescription were significantly higher among patients than matched comparators.

**Figure S5**

Figure S5 Risk of second redeemed A) any psychotropic drug (PD, antidepressant, anxiolytic, or antipsychotic), B) antidepressant, C) antipsychotic, and D) anxiolytic prescription between patients with mastocytosis and matched comparators. Greys test was used to investigate differences in cumulative incidences between patients and comparators. The cumulative incidences of any PD, and antidepressant prescription were significantly higher among patients than matched comparators.

**Figure S6:**

Figure S6: Risk of first redeemed psychotropic drug (PD, antidepressant, anxiolytic, or antipsychotic) prescription after exclusion of hydroxyzine from anxiolytics. Index were defined as five years before the diagnosis date for patients with mastocytosis. Greys test was used to investigate differences in cumulative incidences between patients and comparators. The cumulative incidence of any PD rose more in the time after index among patients. The cumulative incidence of anxiolytics was slightly higher among patients before diagnosis but differed even more between groups in the time after diagnosis.

**Figure S7:**

Figure S7 Risk of first A) contact with psychiatric hospital department regarding anxiety or depression, B) contact with private tax-funded psychologist/psychiatrist not restricted on cause, and C) intentional self-harm or suicidal event among patients with mastocytosis and matched comparators. Greys test was used to investigate differences in cumulative incidences between patients and comparators. No significant difference was observed between patients and matched comparators.

**Figure S8**

Figure S8 Risk of first contact with any psychiatric hospital department among patients with mastocytosis and matched comparators. Greys test was used to investigate differences in cumulative incidences between patients and comparators. No significant difference was observed between patients and matched comparators.
